# COVID-19 Vaccine Uptake by Infection Status in New South Wales, Australia

**DOI:** 10.3201/eid2905.230047

**Published:** 2023-05

**Authors:** Heather F. Gidding, Sandrine Stepien, Jiahui Qian, Kristine K. Macartney, Bette Liu

**Affiliations:** University of Sydney Northern Clinical School, St. Leonards, New South Wales, Australia (H.F. Gidding);; National Centre for Immunisation Research and Surveillance, Westmead, New South Wales, Australia (H.F. Gidding, S. Stepien, J. Qian, K.K. Macartney, B. Liu);; University of New South Wales School of Population Health, Kensington, New South Wales, Australia (H.F. Gidding, J. Qian, B. Liu);; University of Sydney Faculty of Medicine and Health, Camperdown, New South Wales, Australia (H.F. Gidding, K.K. Macartney)

**Keywords:** COVID-19, coronavirus disease, severe acute respiratory syndrome coronavirus 2, SARS-CoV-2, viruses, respiratory infections, data linkage, vaccination, Australia

## Abstract

Using linked public health data from Australia to measure uptake of COVID-19 vaccination by infection status, we found coverage considerably lower among infected than uninfected persons for all ages. Increasing uptake of scheduled doses, including among previously infected persons after the recommended postinfection delay, is needed to reduce COVID-19 illness rates.

Although coverage with 2 doses of COVID-19 vaccine rapidly reached >95% in adults in Australia by late 2021 (*1*), by December 4, 2022, uptake had slowed and plateaued at much lower levels for 2 doses among children 5–15 years of age (52.1%) and for boosters among adults (72.4% for dose 3 and 44.3% for dose 4) (*1*). At the time of this analysis, deferring a scheduled COVID-19 vaccination by 3 months after SARS-CoV-2 infection was recommended; that period has now been changed to 6 months (*2*,*3*). Because a history of infection can influence perceptions about protection against and risk of future infection, we aimed to examine whether timing and uptake of vaccination were affected among persons with recent SARS-CoV-2 infection. We obtained ethics approval for this study from the New South Wales (Australia) Population Health Services Research Ethics Committee (project 2022/ETH00584). 

We linked Australian Immunisation Register (AIR) data and COVID-19 notifications for residents ≥5 years of age on January 1, 2022, living in either the Greater Sydney Metropolitan or Hunter New England areas of New South Wales. AIR includes data on COVID-19 vaccine receipt (by vaccination date, brand, and dose number) among persons registered on Medicare, Australia’s national health insurance program, and for unregistered persons who reported having received a COVID-19 vaccine. Reporting COVID vaccinations to AIR and positive COVID-19 PCR or rapid antigen test results to public health authorities was mandatory during the study period. Study data were available through May 29, 2022. 

We calculated the cumulative percentages of study participants who received the next recommended COVID-19 vaccine dose by infection status and by time after the current dose (i.e., dose 1 for the 5–11 and 12–15 year age groups; dose 2 for the 16–39, 40–64, and ≥65 year age groups; and dose 3 for ≥65 year age group) (Figure). We based infection status on data from COVID-19 notifications as follows: no infection before receiving the next scheduled dose or, if the person did not receive the next dose, by the end of follow-up; infected before current dose; infected after current dose but before the due date for the next dose; or infected after exceeding the due date for the next dose (Table). 

**Figure:**
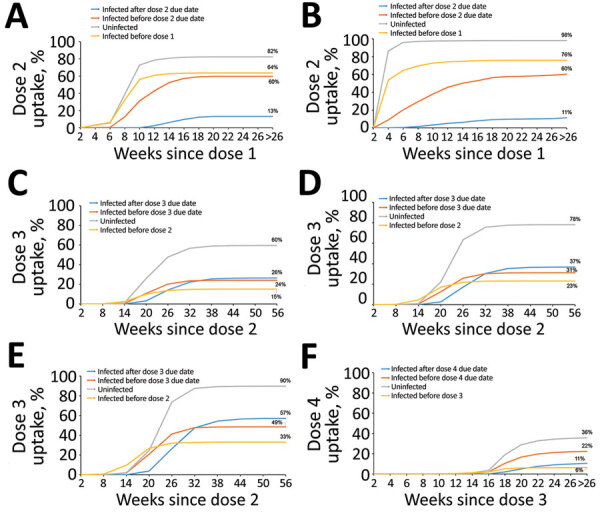
Cumulative uptake of next dose by time since current dose in study of COVID-19 vaccine uptake, by age group and infection status as at May 29, 2022, Greater Sydney Metropolitan and Hunter New England areas of New South Wales, Australia. A, B) Dose 2 uptake for the 5–11-year (A) and 12–15-year (B) age groups; C–E) dose 3 uptake for the 16–39-year (C), 40–64-year (D), and ≥65-year (E) age groups; F) dose 4 uptake for the ≥65-year age group.

**Table:** Age and dose specific cohorts and their distribution in study of COVID-19 vaccine uptake, by infection status on May 29, 2022, Greater Sydney Metropolitan and Hunter New England areas, New South Wales, Australia

Age group, y	Current dose*	Next dose*	Recommended time between current and next dose, d	Total cohort	Uninfected, no. (%)	Infected, no. (%)		
						Before current dose	Before next dose due	After next dose due
5–11	Dose 1	Dose 2	63†	285,638	210,004 (73.5)	18,611 (6.5)	49,888 (17.5)	7,135 (2.5)
12–15	Dose 1	Dose 2	28‡	241,490	236,900 (98.1)	1,962 (0.8)	889 (0.4)	1,739 (0.7)
16–39	Dose 2	Dose 3	91§	1864,335	1,413,329 (75.8)	15,345 (0.8)	68,578 (3.7)	367,083 (19.7)
40–64	Dose 2	Dose 3	91§	1,727,123	1,503,582 (87.1)	7,968 (0.5)	21,456 (1.2)	194,117 (11.2)
≥65	Dose 2	Dose 3	91§	885,564	841,931 (95.1)	1,689 (0.2)	5,544 (0.6)	36,400 (4.1)
	Dose 3	Dose 4	91§	779,649	679,155 (87.1)	24,042 (3.1)	42,464 (5.4)	33,988 (4.4)

*Cohorts were assembled based on the current dose under consideration and retrospectively followed up to determine if they received the next dose. †Children 5–11 y of age: recommended interval between doses 1 and 2 was 8 wk so dose 2 due date was set at 9 wk (63 d). ‡Most children 12–15 y of age: recommended interval between doses 1 and 2 was 3 wk so the dose 2 due date was set at 4 wk (28 d). §For persons ≥16 y of age: recommended interval between doses 2 and 3 changed from November 2021 to January 2022 to 3 mo so the dose 3 due date was set at 91 d; 3 mo was also the most recently recommended interval between doses 3 and 4.

Most study participants were uninfected, but distribution by infection status varied by cohort (Table). In all cohorts, vaccine uptake was most rapid and coverage plateaued at the highest levels among uninfected participants, but the level at which coverage plateaued among uninfected participants differed by cohort (range 36%–98%) (Figure). Even after accounting for the recommended 3-month delay between infection and vaccination, we found coverage among infected persons plateaued at considerably lower levels than among uninfected persons. Among children 5–15 years of age, those infected after the due date for dose 2 had substantially lower uptake (11%–13%) than did the subcohorts infected before dose 1 or between doses 1 and 2 (≥60%). Among all the adult cohorts (>16 years of age), uptake was more similar among the 3 infected subcohorts; coverage plateaued lowest (range 6%–33%) among those infected before the current dose. 

Our use of population-level data was a primary strength of this study. Two international studies reporting on whether SARS-CoV-2 infection status influences vaccination uptake levels were cross-sectional surveys with low response rates or performed among only healthcare workers (*4*,*5*). Unlike this study, those studies did not examine cumulative vaccine uptake by timing of infection, but they also found previous SARS-CoV-2 infection was associated with lower vaccination uptake. 

Our study was limited by a lack of information on reasons for vaccination decisions, and data were available only through May 29, 2022. In addition, COVID-19 notifications were not linked to national public health databases before June 2021, although relatively few infections occurred in New South Wales before that period (*6*); data only represent infections reported. Although reporting positive PCR and rapid antigen tests was mandatory during the study period, positive results were likely underreported. In addition, persons more likely to be tested and have results reported might also have been more likely to get vaccinated, possibly resulting in an underestimation of true discrepancies among subcohorts. 

In conclusion, our study shows that persons with previous SARS-CoV-2 infection were less likely to take up subsequent recommended vaccine doses than uninfected persons. Because previous infection alone is unlikely to provide sufficient protection against severe disease (*2*), greater adherence to vaccine recommendations is required to reduce health effects from COVID-19. Ongoing monitoring of vaccination uptake and timely linkage to infection status could help better understand gaps between SARS-CoV-2 population immunity and vaccine recommendations. Those data, together with information from surveys to identify drivers of delayed vaccination among infected populations, would enable development of appropriately targeted public health campaigns to reduce COVID-19–related illness rates. 
